# PLC-Based Integrated Refractive Index Sensor Probe with Partially Exposed Waveguide

**DOI:** 10.3390/s22155672

**Published:** 2022-07-29

**Authors:** Jin Hwa Ryu, Hoesung Yang, Soyoung Park, Soocheol Kim, Kyuwon Han, Hyunseok Kim, Kwangsoo Cho, Kang Bok Lee

**Affiliations:** Industrial & Personal Safety Intelligence Research Section, Electronics and Telecommunications Research Institute (ETRI), Daejeon 34129, Korea; hsyang@etri.re.kr (H.Y.); bubble@etri.re.kr (S.P.); skim88@etri.re.kr (S.K.); wally.han@etri.re.kr (K.H.); hyunseok@etri.re.kr (H.K.); choks@etri.re.kr (K.C.); kblee@etri.re.kr (K.B.L.)

**Keywords:** integrated PLC probe, refractive index sensor, U-shaped waveguide, trench, index contrast

## Abstract

This paper proposes a simple, high-efficiency refractive index (RI) sensor, with a structure based on the planar lightwave circuit (PLC) probe type. The optical sensor has a 1 × 2 splitter structure with reference and sensing channels, each consisting of a U-shaped waveguide structure that is configured by connecting C bends. This design allows for the sensor device to have a probe structure wherein the surface interconnected with activity devices (i.e., an optical source and optical detector) is placed on one side. The reference channel is bent with a minimum optical loss, and the sensing channel has a bent structure, involving a C-bend waveguide with a maximum loss. The C-bend waveguide with a maximum loss is conformally aligned to have a trench structure with the same bending radius, designed to selectively expose the sidewall of the core layer. The local index contrast varies depending on the material in contact with the trench, resulting in a change in the optical output power of the waveguide. The sensitivity of the proposed sensor was 0 and 2070 μW/refractive index unit (RIU) for the reference and sensing channels, respectively, as the RI changed from 1.385 to 1.445 at a 1550 nm wavelength. These results suggest that the proposed structure enables efficient RI measurement through the use of a simple dip-type method.

## 1. Introduction

The refractive index (RI) is an inherent optical property of a given material, and precise techniques are required for its measurement in various fields, such as safety, biomedical, medical, chemical, and environmental monitoring. To this end, various studies have been conducted on the geometrical structure and material design of optical devices, such as the plasmonic-effect-based [1,2], silicon slot [3], liquid-crystal cladding [4], Mach Zehnder interferometer [5,6], multimode double-cladding multimode [7], and tapered-structure-based [8] waveguides. Optical-fiber-based devices have been investigated because of their low cost and ease of use; however, they have limitations concerning their integration and mass production. Technology based on planar lightwave circuits (PLCs) have been studied as an alternative. PLC devices allow for the quantitative control of the variables, which determines the characteristics of an optical device, including the structure, dimension, bending, material, and RI difference, and this technology can be highly integrated in mass-producing semiconductor processes. PLCs are an active field of research because the characteristics of these sensors, such as sensitivity and range, can be quantitatively designed while ensuring the sensor’s stability and reliability. Although optical sensor technology has made considerable progress in recent times, it still has limitations in improving its practicality in applications such as simple point-of-care testing (POCT).

To improve the practical applications of optical sensors, sensor technology must be both highly integrated and efficient. For this purpose, probe-structure-based optical sensor technology has been studied [9,10,11,12,13,14,15,16,17]. The sensor requires the surface to be placed on a single side where the optical source and detector are interconnected; in many cases, this causes an abrupt change in the direction of the optical path. In such cases, bend loss occurs in dielectric waveguides. Studies have been conducted on various high integrated optical devices to overcome bend loss, including air trenched, graded-index cores, and hybrid waveguide structures [18,19,20]. Among these methods, the difference in the RI between the core and clad can be used as a variable to control the light confinement characteristics. A decrease in the bending radius of the optical waveguide leads to an increase in the radiation loss and evanescent field. At the same time, the index contrast (Δ*n*) acts as a variable that controls the optical characteristics; with decreasing bending radius, the higher the sensitivity to changes in the propagated output power of the optical waveguide with variations in the RI contrast [21,22].

The aim of this study was to propose a highly efficient sensor structure and verify its effectiveness. The proposed optical sensor device incorporates a highly sensitive, dip-type sensor probe structure that is based on a bent waveguide with minimal optical propagating characteristics.

## 2. Concepts Regarding the Integrated PLC Sensor Probe

**Figure 1:**
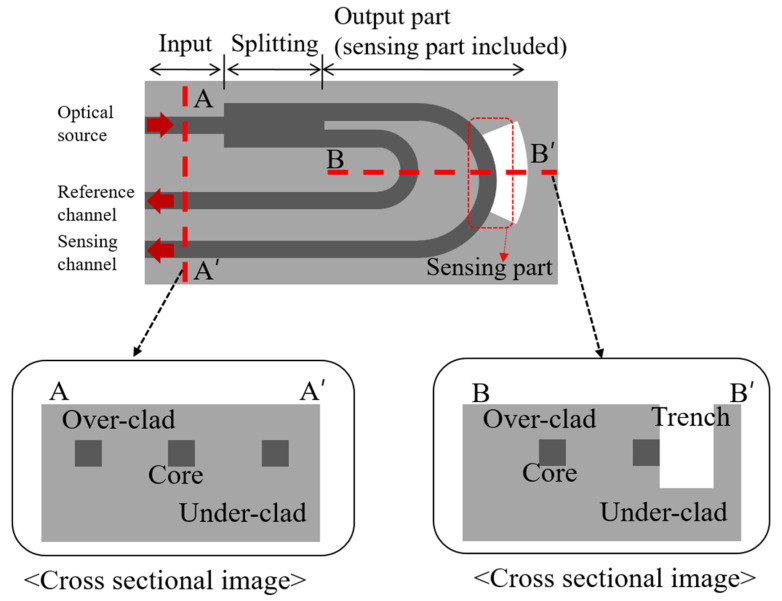
Schematic configuration of an integrated planar lightwave circuit (PLC) sensor probe.

**Figure 2:**
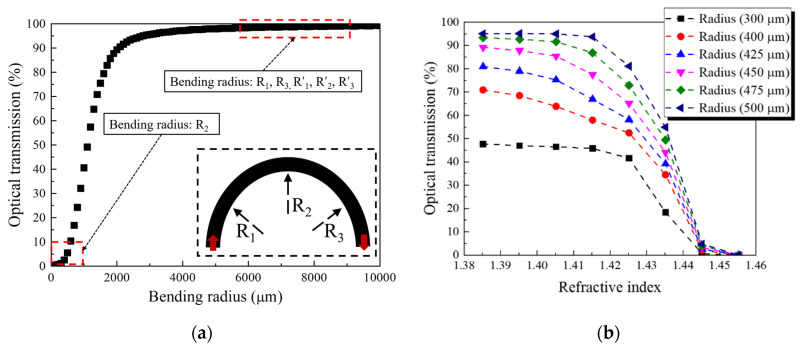
Optical characteristics (simulation results) of the bent waveguide, according to (**a**) bending radius (inset: connected C-bend structures of the U-shaped waveguide), and (**b**) variation in the refractive index (RI) of the trench.

We propose a highly efficient RI sensor with a dip-type probe structure, based on a U-shaped waveguide (Figure 1). The sensor has a 1 × 2 splitter structure, consisting of an input port (through which the optical source is launched), splitting part, and output ports with reference and sensing channels. In the PLC probe structure, the channels of the input and output ports that are interconnected with the active devices (i.e., optical source and optical detector) are placed on one side (cross-section A in Figure 1). The channels of the output ports are bent into a U-shaped structure by connecting C-bend structures with various bending radii. The reference channel maintains stable optical waveguide characteristics, but the sensing channel has a trench structure, where the sidewall of the core layer is selectively exposed (no cladding). The waveguide characteristics vary depending on the external material in contact with the trench (cross-section B in Figure 1). Specifically, the local index contrast changes depending on the material in contact with the trench area. The U-shaped structure was configured by introducing a bent waveguide, which was designed and optimized using the beam propagation method (BPM) to achieve high sensitivity and high integration. The optical device was designed as a 6 × 6 μm single-mode waveguide, and the RI difference was 0.75% (clad RI: 1.444) at 1550 nm. The launched optical source was designed to divide into the output port channels at a ratio of 50:50, and the bending radii for the output port channels were subsequently designed. A BPM-based simulation showing the results of the optical sensor is depicted in Figure 2. The theoretical details of this simulation and subsequent results were described in our previous study [21]. Figure 2a shows the optical waveguide characteristics according to the bending radius. The U-bend structure was configured by connecting three C. bends. The reference channel was configured by connecting three C bends with the same bending radius of 6000 μm (R′_1_ = R′_2_ = R′_3_), having 98% propagation characteristics in terms of integration. The sensing channel was configured by connecting two C bends (R_1_ = R_3_) with a bending radius of 8400 μm, which would allow the channel to maintain at least 99% of the propagation characteristics, and one C bend (R_2_) with a maximum loss in terms of the improved sensitivity. The trench structure was conformally aligned to have the same bending radius as the waveguide, with a bending radius of R_2_. Figure 2b shows the optical characteristics of the bent waveguide according to the variation in the RI of the trench, which we conducted to verify the sensitivity and effectiveness of the sensor. The optical characteristics were examined as the RI of the trench varied from 1.385 to 1.455 based on a bent waveguide, with an optical propagating characteristic of less than 5%. The optical characteristics improved as the RI of the trench decreased relative to the reference RI value of 1.444, demonstrating the effectiveness of the sensor. Moreover, as the bending radius increased from 300 to 500 μm, the optical characteristics improved. However, no change in sensitivity was observed in the high RI difference at a bending radius of 475 μm or more. Therefore, the R_2_ bending radius was set to 450 μm to achieve high sensitivity.

## 3. Materials and Methods

**Figure 3:**
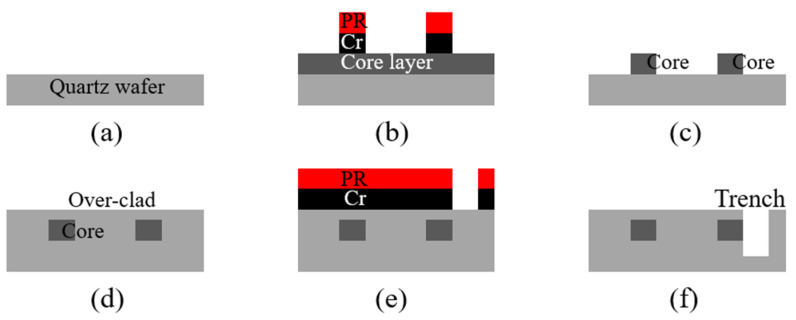
Schematic of the fabrication procedure for the integrated PLC sensor probe device: (**a**) fused-synthesis quartz wafer; (**b**) fabrication of the core structure; (**c**) fabricated core structure; (**d**) fabrication of over-clad; (**e**) fabrication of the trench structure; and (**f**) fabricated PLC sensor probe device.

The PLC sensor probe device was fabricated using two-step photolithography based on the 6-inch quartz wafer process. Figure 3 shows a schematic of the fabrication process. The overall procedure consists of two steps: First, fabricate a PLC device embedded with a waveguide, having a maximum loss and a uniform RI difference between the core and cladding. Second, fabricate a sensor probe device with an area that has a varying local index contrast due to the trench.

The first PLC device fabrication was performed by using a fused-synthesis quartz wafer (RI: 1.444 at 1550 nm). A core layer with a difference in the RI of 0.75% was prepared by depositing germanium-doped SiO_2_ at a thickness of 6 μm through a plasma-enhanced chemical vapor deposition (PECVD) process, followed by heat treatment at 1240 °C for 12 h. The 1 × 2 splitter core structure was constructed by etching at a depth of 6.6 μm using an inductively coupled plasma reactive ion etching (ICPRIE) process. A 200 nm thick Cr layer was prepared by the DC sputtering process and used as the etching mask. Finally, the over-clad was fabricated to have a thickness of 13 μm through a flame hydrolysis deposition process at a humidity of 10.7%, and heat treatment was conducted at a temperature of 1200 ℃ for 12 h.

**Figure 4:**
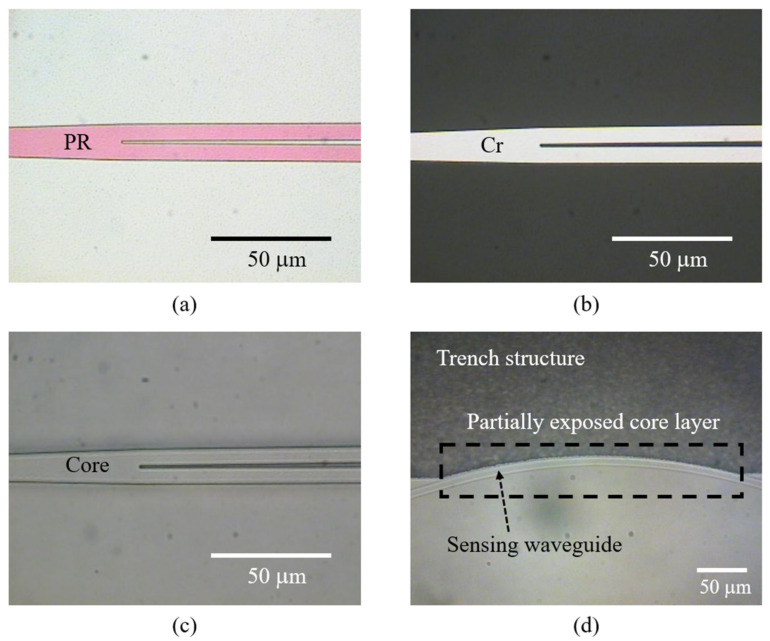
Optical images of the integrated PLC sensor probe device following the fabrication procedure: (**a**) photoresist pattern; (**b**) Cr mask pattern; (**c**) core pattern; (**d**) conformal aligned trench structure.

The second sensor probe device fabrication was performed to form a trench structure on the sidewall of the core area, with a maximum loss of the PLC device. The trench structure was fabricated to conformally align with the sensing channel having an R_2_ bending radius; an 860 nm thick Cr layer was used as an etching mask to conduct the ICPRIE process, forming a width of 1000 μm and depth of 20 μm. The trench structure was fabricated so that the bending radius would extend to 1000 μm in each area that was not conformally aligned with the sensing channel, to establish a stable external contact medium environment. As a result, only one side of the sensing channel with the R_2_ bending radius was selectively exposed. The results from various stages of the fabrication procedure are shown in Figure 4.

## 4. Results and Discussion

**Figure 5:**
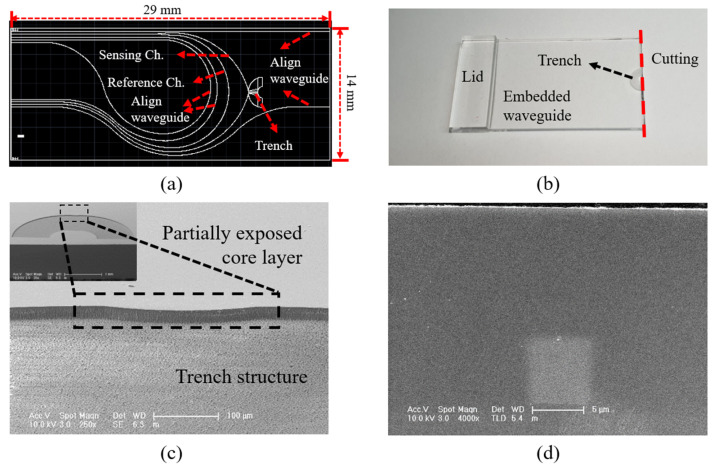
Integrated PLC sensor probe device with the partially exposed core layer: (**a**) PLC device design; (**b**) fabricated PLC device; (**c**) scanning electron microscope (SEM) image of the etched trench structure; (**d**) cross-sectional SEM image of the fabricated PLC device.

Figure 5 shows the fabricated PLC sensor probe device. The design and structure of the PLC device are indicated in Figure 5a. The device has a 1 × 2 splitter structure based on the U-shaped waveguide, and the trench is aligned with the sensing channel at the R_2_ bending radius. In consideration of the optical interconnection, the input and output channels interconnected to the active devices were designed in a half-pitch (127 μm pitch) structure. Figure 5b shows an image of the fabricated PLC device, where the sensing channel is only partially exposed because of the trench structure; the rest of the device has an embedded PLC structure. The sensor device includes only the splitter structure and the trench structure area. Therefore, other areas may be cut. Figure 5c shows a scanning electron microscope (SEM) image of the trench structure, where only the sensing channel with a bending radius of 450 μm is selectively exposed. The overall trench structure was fabricated in a C shape, and a stable contact medium environment was established. Figure 5d is a cross-sectional SEM image of the fabricated PLC device, which exhibits the embedded 6 × 6 μm channel waveguide.

The optical characteristics of the PLC sensor probe were measured using an optical source with a wavelength of 1550 nm, and the sensor was interconnected in a pig-tailed module using a 64-channel fiber array (FA) block with a 127 μm pitch. The characteristics of the interconnected sensor module were measured at 249.46 μW (−6.03 dBm) and 131.83 μW (−8.8 dBm) for the reference and sensing channels, respectively. The characteristic evaluation was performed by measuring the change in the output power intensity of the sensor probe module, resulting in the RI values varying with the change in glycerol concentration. The optical sensor module was structurally designed with a RI range of 1.385 to 1.455. Therefore, the glycerol solution was prepared at a concentration range of 50 to 100 wt%, using deionized water as a dilution solvent. The resulting RI was equivalent to 1.382 to 1.457, based on Equation (1), where *n* is the RI, and *w* is the glycerol concentration [23]:

*n* = −0.0216 *w*^3^ + 0.0512 *w*^2^ + 0.111 *w* + 1.31165 (1)

**Figure 6:**
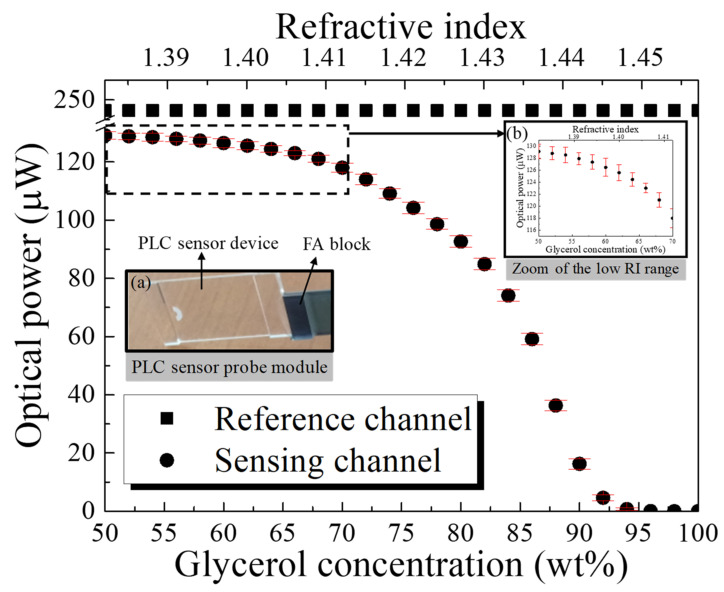
Optical characteristics of the PLC sensor probe, according to the RI. (Inset figures: (**a**) pig-tailed PLC sensor probe module; (**b**) zoom of the low RI range).

The characteristic evaluation of the optical sensor module was performed by increasing the glycerol concentration (G7893, Sigma-Aldrich Co., St. Louis, MO, USA) from 50 wt% in intervals of 2 wt%. Figure 6 shows the results for the fabricated optical sensor module.

The RI was measured three times for the same solution. The rectangular symbols indicate the optical power of the reference channel, which maintained a value of 249.46 μW independent of the change in RI. The circular symbols indicate the average optical power of the sensing channel with respect to the RI, which was found to vary from 129.12 to 0 μW, with a maximum variation of ±2 μW. The insets in Figure 6 show (a) the dip-type PLC sensor probe module, with the surface interconnected to the active devices placed on one side, and (b) the zoom of the low RI range. As the optical sensor module was designed and fabricated to partially expose the core layer with the trench, the optical characteristics increased as the RI of the trench reached a low value relative to the reference RI of 1.444, resulting in a change in the high RI. Conversely, the optical characteristics decreased as the low RI approached the reference RI, resulting in a difference in the low RI. The sensitivity, *S*, can be calculated as *S* = Δ*I*/Δ*n*, where Δ*I* is the optical power intensity variation, and Δ*n* is the change in the RI of the material in contact with the trench. The sensitivity was measured at 1722 μW/refractive index unit (RIU) over the entire variation range of the RI (RI: 1.382 to 1.457), but it was confirmed to be 2070 μW/RIU (optical power: 128.82 μW to 4.6 μW) over the RI range of 1.385 to 1.445, based on the design range of the sensor module and the reference RI. The measurement results of the optical sensor exhibited a specific tendency, depending on the variation in the RI. The change in sensitivity was confirmed to be 222 μW/RIU (optical power: 129.12 to 124.45 μW) over the RI range from 1.382 to 1.403, 1230 μW/RIU (optical power: 124.45 to 98.63 μW) over the RI range from 1.403 to 1.424, and 4478 μW/RIU (optical power: 98.63 to 4.6 μW) over the RI range from 1.424 to 1.445. Based on the RI of 1.444, the change in sensitivity decreased with a change in the high RI. Conversely, the change in sensitivity increased with a change in the low RI. Additionally, a sensitivity of 383 μW/RIU (optical power: 4.6 to 0 μW) was observed in the RI range from 1.445 to 1.457, which exceeds the reference RI. This is because the total internal reflection (TIR) condition was not satisfied, owing to the inclusion of the range exceeding the RI of the core, resulting in an area where a total loss would occur. The effectiveness of the proposed sensor probe module was confirmed, based on the measurement results and the structurally designed simulation results of the optical sensor (the optical characteristic of the bending radius of 450 μm is shown in Figure 2b).

## 5. Conclusions

This study was conducted with the aim of proposing a dip-type PLC sensor probe with high efficiency. The sensor probe was designed to achieve high sensitivity by placing the surface interconnected with the active devices on a single side and aligning the trench structure in a partial section of the bent waveguide with maximum optical loss. The PLC sensor probe was configured as a 1 × 2 splitter device having a U-shaped waveguide, and its effectiveness was verified by interconnecting it with a FA block and measuring the change in the resulting optical output from the variation of the RI.

Over the RI range of 1.385 to 1.445 in the fabricated sensor probe, the reference channel demonstrated a uniform optical output of 249.46 μW independent of the change in RI, whereas the sensing channel demonstrated an optical output of 128.82 to 4.6 μW and a sensitivity of 2070 μW/RIU in response to the changing RI. Although this study was only conducted to verify the proposed sensor probe, it is also possible to control characteristics such as the range, sensitivity, and linearity of sensing results by varying the design of the optical source wavelength and the waveguide structure (splitting ratio, radius, etc.). Moreover, the sensor can be applied as a gas sensor by placing a functional material that is reactive with gas particles, such as a liquid crystal and semiconductor metal oxide, in the trench area. Therefore, the sensor is expected to have applications for multiple purposes and fields.

## Data Availability

Not applicable.
